# High incidence of lung cancer death after curative endoscopic submucosal dissection for superficial esophageal squamous cell carcinoma

**DOI:** 10.1002/cam4.7242

**Published:** 2024-05-11

**Authors:** Ayaka Tajiri, Yoshiki Tsujii, Tsutomu Nishida, Takuya Inoue, Akira Maekawa, Shinji Kitamura, Shinjiro Yamaguchi, Akihiro Nishihara, Takuya Yamada, Hideharu Ogiyama, Yoko Murayama, Shunsuke Yamamoto, Satoshi Egawa, Ryotaro Uema, Takeo Yoshihara, Yoshito Hayashi, Tetsuo Takehara

**Affiliations:** ^1^ Department of Gastroenterology and Hepatology Osaka University Graduate School of Medicine Suita Japan; ^2^ Department of Gastroenterology Toyonaka Municipal Hospital Toyonaka Japan; ^3^ Department of Gastroenterology Osaka General Medical Center Osaka Japan; ^4^ Department of Gastroenterology Osaka Police Hospital Osaka Japan; ^5^ Department of Gastroenterology Sakai City Medical Center Sakai Japan; ^6^ Department of Gastroenterology Kansai Rosai Hospital Amagasaki Hyogo Japan; ^7^ Department of Gastroenterology Minoh City Hospital Minoh Japan; ^8^ Department of Gastroenterology Osaka Rosai Hospital Sakai Japan; ^9^ Department of Gastroenterology Ikeda City Hospital Ikeda Japan; ^10^ Departments of Gastroenterology and Hepatology Itami City Hospital Itami Hyogo Japan; ^11^ Department of Gastroenterology and Hepatology National Hospital Organization Osaka National Hospital Osaka Japan; ^12^ Department of Gastroenterology Kinki Central Hospital of Mutual Aid Association of Public School Teachers Itami Hyogo Japan

**Keywords:** endoscopic submucosal dissection, esophageal squamous cell cancer, lung cancer, second primary cancer

## Abstract

**Background and Aim:**

Following treatment of superficial esophageal squamous cell carcinoma (ESCC), surveillance for a second primary malignancy (SPM) is necessary. However, detailed evidence regarding the timing and prognosis of SPMs is insufficient. We aimed to clarify the details of SPMs and their effects on patient outcomes.

**Methods:**

This retrospective, multicenter study involved 11 hospitals. Patients with superficial ESCC curatively resected using endoscopic submucosal dissection between May 2005 and December 2012, were included in this study.

**Results:**

The 5‐year survival rate of 187 patients was 92.6% during a median follow‐up duration of 96.8 months. Thirty‐one patients died, 14 of whom died of SPMs. Compared to patients with SPMs detectable by esophagogastroduodenoscopy (EGD), patients with SPMs detectable only by modalities other than EGD had a significantly higher mortality rate (*p* < 0.001). Patients with second primary lung cancer (LC) had a high mortality rate (56.3%). Univariate and multivariate analyses showed that multiple Lugol‐voiding lesions (LVLs) tended to be associated with SPMs (*p* = 0.077, hazard ratio [HR] 4.43, 95% confidence interval [CI]: 0.91–6.50), and metachronous ESCC was an independent risk factor for the incidence of second primary LC (*p* = 0.037, HR 3.51, 95% CI: 1.08–11.41).

**Conclusions:**

SPMs that cannot be detected by EGD, such as LC, must be considered after the curative resection of ESCC. We suggest strict screening by both EGD and computed tomography for patients with multiple LVLs or metachronous ESCC to detect SPMs in their early stages.

## Graphical abstract

Patients after treatment of superficial esophageal squamous cell carcinoma (ESCC) had a worse outcome if they had second primary malignancies (SPMs). Especially, patients with second primary lung cancer (LC) had a high mortality rate (56.3%). Multiple Lugol‐voiding lesions tended to be associated with SPMs (*p* = 0.077, hazard ratio [HR] 4.43, 95% confidence interval [CI]: 0.91–6.50), and metachronous ESCC was an independent risk factor for the incidence of second primary LC (*p* = 0.037, HR 3.51, 95% CI: 1.08–11.41).

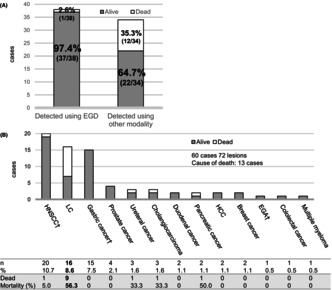

## 1 INTRODUCTION

Esophageal cancer (EC) is the seventh most common cancer, and squamous cell carcinoma (SCC) is the most common histological subtype, accounting for 80% of all EC cases worldwide.^1^ EC is the sixth most common cause of cancer‐related deaths.^1^ If EC is detected and treated at an early stage, the outcomes may be favorable. Specifically for superficial EC, minimally invasive and curative treatments such as endoscopic resection (ER) techniques, including endoscopic submucosal dissection (ESD) and endoscopic mucosal resection (EMR), have significantly improved prognosis while ensuring safety.^2^, ^3^, ^4^ Esophageal squamous cell carcinoma (ESCC) diagnosed as T1a based on the invasion of epithelium/lamina propria mucosae (EP/LPM), is considered to be cured due to its minimal risk of metastasis (0–0.4%).^5^, ^6^, ^7^, ^8^, ^9^ Even in cases where it invades the muscularis mucosa (MM), the risk of metastasis is relatively low (0–4.3%) with the absence of lymphovascular involvement and negative pathological margins.^8^, ^10^, ^11^ Therefore, considering the risk and invasiveness, the Esophageal Cancer Practice Guidelines (2022)^9^ allow for the observation of such cases without additional treatments, such as chemoradiotherapy (CRT) or surgery, considering the risk and invasiveness.

Surveillance esophagogastroduodenoscopy (EGD) is necessary after ER for the early detection of metachronous ESCC.^4^, ^9^ In addition, the ESD/EMR guidelines for EC in Japan^12^ mention the need for surveillance of second primary malignancies (SPMs) in other organs. Previous studies have shown a high risk of other malignancies after ESCC treatment^13^, ^14^, ^15^, ^16^ and their effects on long‐term outcomes.^7^, ^17^ However, there is currently insufficient evidence to determine when other malignancies are detected or whether they are the cause of death in cases of curative ESCC resection. Therefore, this study aimed to clarify the details of SPMs, including their organs of origin or timing of detection, and how they affect patients' long‐term outcomes after curative resection of superficial ESCC.

## 2 METHODS

### 2.1 Study design and patients

This multicenter retrospective study included 11 hospitals (one university hospital and 10 secondary or tertiary care hospitals; Table S1) that had participated in our previous study.^4^ From the databases and medical records of each institute, we identified and enrolled patients with esophageal neoplasia (ESCC or suspected ESCC) who had undergone ESD treatment between May 2005 and December 2012. The inclusion criteria for patients were as follows: (1) ESCC with a pathological depth of invasion up to the EP/LPM or MM, or high‐grade intraepithelial neoplasia (HGIN), indicating lesions involving more than half of the epithelium; (2) no lymphovascular invasion;(3) negative horizontal and vertical margins; and (4) ESD was performed for the first time. We included “HGIN” cases, despite the outdated terminology, as these cases encompassed, cancer in situ as previously defined.^18^, ^19^, ^20^ The exclusion criterion was a history of treatment other than ESD, such as surgery or CRT, for EC.

### 2.2 Clinical information

We retrospectively collected patient information, including background factors such as sex, age, history of other organ malignancies, alcohol consumption, and smoking, and endoscopic findings such as lesion size, location, macroscopic type, and pathological depth of invasion. The incidence of metachronous ESCC and SPMs and the long‐term prognosis, especially in relation to SPMs, were investigated. SPMs were defined as malignancies diagnosed more than 2 months after the initial primary ESCC diagnosis in accordance with the SEER rules of the National Cancer Institute.^21^ It did not include metachronous ESCC. The decision whether to screen for other organ malignancies before ESD depended on each physician.

### 2.3 Histological evaluation and follow‐up

The specimens resected by ESD were fixed in 10% formalin and cut into 2‐mm‐thick slices. Histological assessment of the specimens was conducted according to the Japanese Classification of EC.^22^ The size, histology, and invasion depth of the pathologically diagnosed specimens were analyzed.

The interval between post‐ESD surveillance EGDs was determined by each physician based on the patient's background, such as age and comorbidities; however, follow‐up EGD were typically conducted every 6–12 months. No specific guidelines were in place for performing computed tomography (CT) scans since the lesions were endoscopically diagnosed as superficial ESCC without submucosal invasion and histologically confirmed as curatively resected. Therefore, CT scans were performed approximately once a year, when each physician considered it necessary.

### 2.4 Statistical analysis

Continuous variables are presented as median (range), while categorical variables are summarized as frequencies (percentages). Continuous variables were compared using the Wilcoxon test and categorical variables were compared using χ^2^ or Fisher's exact tests. The development of metachronous ESCC and SPMs and survival were analyzed using the Kaplan–Meier method and the log‐rank test. For some SPMs, we calculated the standardized incidence ratios (SIRs) using indirect standardization methods based on the Cancer Statistics of Japan (Cancer Statistics, Cancer Information Service, National Cancer Center, Japan [National Cancer Registry, Ministry of Health, Labor, and Welfare]).^14^, ^23^ The data for 2020 and beyond were yet unpublished; therefore, we substituted them with those of 2019. The exact confidence intervals (CIs) around the SIR were calculated assuming a Poisson distribution of the observed number of neoplasms. We identified the factors related to SPMs using a Cox proportional hazards model with time‐dependent covariates for univariate and multivariate analyses, estimating the hazard ratio (HR) and 95% CI. The adjustment was made for the SPMs and metachronous ESCC occurring after the first treatment for ESCC. Statistical significance was defined as *p* < 0.05. All analyses were conducted using EZR software (version 1.54, Saitama Medical Center, Jichi Medical University, Tochigi, Japan) on a personal computer.

### 2.5 Ethics approval

The study protocol was approved by the Institutional Review Boards (IRB) of Osaka University (no. 16352) and other participating institutions. The study was conducted in accordance with the principles of the Declaration of Helsinki. All participants were given the opportunity to decline participation in the study before the current investigation, utilizing the opt‐out method made available on each hospital's website. An exemption of written informed consent was granted by the IRB for this purpose.

## 3 RESULTS

### 3.1 Patients and lesions

During the study period, 312 patients underwent ESD for superficial esophageal neoplasia. Among them, a total of 187 patients who met the inclusion and exclusion criteria were enrolled in this study. The patient and lesion characteristics are shown in Table 1. Metachronous ESCC (25.7%) and SPMs (32.1%) developed during a median follow‐up period of 96.8 months (range: 4.6–192.7 months) after curative resection of ESCC.

**TABLE 1:** Patient and lesion characteristics.

All patients	187
Age, median [range], years	69 [49–92]
Sex	
Male	150 (80.2%)
Female	37 (19.8%)
Alcohol consumption	
Yes	143 (76.5%)
No	27 (14.4%)
Unknown	17 (9.1%)
Smoking	
Yes	138 (73.8%)
No	33 (17.6%)
Unknown	16 (8.6%)
Chest radiography before ESD	
Yes	158 (84.5%)
No	24 (12.8%)
Unknown	5 (2.7%)
Chest CT scan before ESD	
Yes	145 (77.5%)
No	37 (19.8%)
Unknown	5 (2.7%)
Lesions	
Multiple LVLs	
Yes	150 (80.2%)
No	36 (19.3%)
Unknown	1 (0.5%)
Tumor length, median [range], mm	15 [2–85]
Location	
Cervical‐upper thoracic	29 (15.5%)
Middle thoracic	125 (66.8%)
Lower thoracic‐abdominal	33 (17.6%)
Macroscopic type	
Flat or depressed type (0‐IIb, 0‐IIc)	175 (93.6%)
Elevated (0‐I, 0‐IIa) or Mixed type	12 (6.4%)
Pathological depth of invasion	
HGIN	58 (31.0%)
EP/LPM	103 (55.1%)
MM	26 (13.9%)
Multiple ESCC	
Yes	24 (12.8%)
No	163 (87.2%)
Metachronous malignancies	
Observation time, median (range), months	96.8 [4.6–192.7]
Metachronous ESCC	
Yes	48 (25.7%)
No	139 (74.3%)
Second primary malignancy	
Yes	60 (32.1%)
No	127 (67.9%)

Abbreviations: CT, computed tomography; EP, epithelium; ESCC, esophageal squamous cell carcinoma; ESD, endoscopic submucosal dissection; HGIN, high‐grade intraepithelial neoplasia; LPM, lamina propria; LVL, Lugol‐voiding lesion; MM, muscularis mucosa.

### 3.2 Long‐term outcomes and cause of death

**FIGURE 1:**
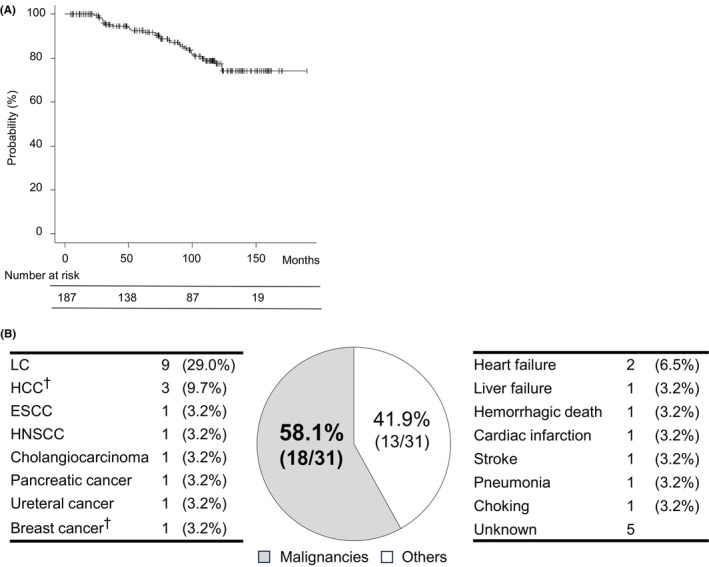
(A) Long‐term outcomes of patients with esophageal cancer after treatment and the details of the cause of death. The Kaplan–Meier curve shows the OS rate. (B) Causes of death in this study. ^†^Malignancies diagnosed before ESCC treatment. ESCC, esophageal squamous cell carcinoma; HCC, hepatocellular carcinoma; HNSCC, head and neck squamous cell carcinoma; LC, lung cancer; OS, overall survival; SPM, second primary malignancy.

Figure 1A shows the long‐term outcomes of superficial ESCC resections. The 5‐year survival rate was 92.6% and 31 deaths occurred. The details of the causes of death are shown in Figure 1B. Eighteen of the deaths (58.1%) were malignancy‐related, and 14 of these (77.8%) occurred after ESCC treatment. There was only one case of ESCC‐induced death, which was EP/LPM with metastasis to the lymph nodes and was treated with CRT.

### 3.3 SPMs and their influences on the long‐term outcome

**FIGURE 2:**
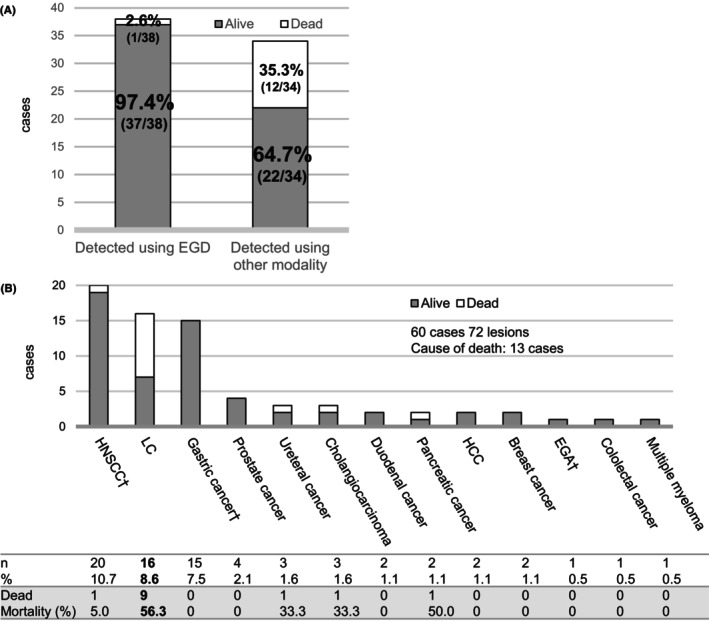
(A) Mortality rate and second primary malignancy stratified by the modality of detection. Patients whose SPM was detected using a modality other than EGD showed significantly higher mortality rates than those whose SPM was detected using EGD (*p* < 0.001). (B) Mortality rate and second primary malignancies. Although EGA is also an esophageal cancer, we considered it a distinct cancer from ESCC because it originates from gastric mucosa and has different risk factors. ^†^Malignancies detected using EGD. EGA, esophagogastric adenocarcinoma; EGD, esophagogastroduodenoscopy; ESCC, esophageal squamous cell carcinoma; HCC, hepatocellular carcinoma; HNSCC, head and neck squamous cell carcinoma; LC, lung cancer; SPM, second primary malignancy.

SPMs were divided into two groups: those that can be detected using EGD, occurring from the oral cavity to the duodenum, and those that can be detected using only other modalities, mainly CT scan (Figure 2A). Patients with SPM that can be detected using a modality other than EGD had significantly higher mortality rates than patients with SPMs that can be detected using EGD (*p* < 0.001). Figure 2B illustrates the details of this process. The top three SPMs and their SIRs are as follows: 17.92 (95% CI: 13.91–21.92) for head and neck SCC (HNSCC), 2.83 (2.12–3.53) for lung cancer (LC), and 2.39 (1.77–3.01) for gastric cancer. Notably, patients with a second primary LC had a high mortality rate. Of the patients diagnosed with LC after ESCC resection, 56.3% died of LC.

Table 2 shows that the hazards to prognosis of SPMs, especially second primary LCs, were significantly high (*p* < 0.001, HR 6.57, 95% CI: 3.10–13.93 and *p* < 0.001, HR 13.03, 95% CI: 6.13–27.67, respectively). We categorized the patients into two groups, those with and without SPMs, to compare their prognoses with the Kaplan–Meier curve (Figure S1A,B). Although they did not correspond to the results of the previous Cox proportional hazard model with time‐dependent covariates, they showed worse prognoses of patients with SPM and second primary LC.

**TABLE 2:** Association between SPM or second primary LC and their prognoses.

	HR for mortality [95% CI]	*p* Value
With SPM		
No	1	<0.001
Yes	6.57 [3.10–13.93]	
With second primary LC		
No	1	<0.001
Yes	13.03 [6.13–27.67]	

Abbreviations: CI, confidence interval; HR, hazard ratio; LC, lung cancer; SPM, second primary malignancy.

### 3.4 Risk factors for SPMs and second primary LC after ESCC resection

Univariate and multivariate analyses were performed to explore risk factors for SPMs (Table 3). Univariate analysis revealed that it was associated with multiple Lugol‐voiding lesions (LVLs) (*p* = 0.010) and metachronous ESCC (*p* = 0.035). Multivariate analysis was performed including these two factors and some factors that were considered to be associated with SPMs in patients with ESCC in previous studies.^15^, ^16^, ^24^ Multiple LVLs tended to be associated with the SPM incidence after ESCC resection (*p* = 0.077, HR: 4.43, 95% CI: 0.91–6.50); however, no independent risk factor was revealed.

**TABLE 3:** Univariate and multivariate analyses for second primary malignancies after ESCC resection.

	Second primary malignancies	Univariate analysis HR [95% CI]	*p* Value	Multivariate analysis HR [95% CI]	*p* Value
Age					
<70‐years‐old	32.7% (32/98)	1	0.955	1	0.340
70‐years‐old	31.5% (28/89)	1.01 [0.61–1.70]		1.31 [0.75–2.26]	
Sex					
Female	27.0% (10/37)	1	0.666	1	0.702
Male	33.3% (50/150)	1.16 [0.69–2.29]		0.85 [0.38–1.93]	
Smoking					
No	27.3% (9/33)	1	0.360	1	0.557
Yes	36.2% (50/138)	1.45 [0.71–2.94]		1.27 [0.57–2.82]	
Unknown	6.3% (1/16)				
Alcohol consumption					
No	33.3% (9/27)	1	0.416	1	0.650
Yes	35.0% (50/143)	1.34 [0.66–2.74]		1.22 [0.51–2.93]	
Unknown	5.9% (1/17)				
Multiple LVLs					
No	13.9% (5/36)	1	0.010	1	0.077
Yes	36.0% (54/150)	3.33 [1.33–8.36]		2.43 [0.91–6.50]	
Unknown	100% (1/1)				
Location					
Upper	37.9% (11/29)	1	0.199		
Middle	28.0% (35/125)	0.61 [0.31–1.20]			
Lower	42.4% (14/33)	0.96 [0.44–2.13]			
Size					
<15 mm	34.9% (29/83)	1	0.681		
≥15 mm	29.8% (31/104)	0.90 [0.54–1.49]			
Macroscopic type					
Flat or depressed type	30.9% (54/175)	1	0.228		
Elevated or mixed type	50.0% (6/12)	1.68 [0.72–3.91]			
Pathological Invasion depth					
HGIN	35.6% (26/73)	1	0.591		
EP/LPM	29.6% (26/88)	0.75 [0.44–1.30]			
MM	39.8% (8/26)	0.81 [0.37–1.79]			
Simultaneous or history of cancer in other organs					
No	29.5% (38/129)	1	0.216	1	0.514
Yes	37.9% (22/58)	1.41 [0.83–2.42]		1.20 [0.69–2.07]	
Metachronous ESCC					
No	24.5% (34/139)	1	0.035	1	0.157
Yes	52.1% (25/48)	1.92 [1.05–3.53]		1.60 [0.83–3.07]	

Abbreviations: CI, confidence interval; EP, epithelium; ESCC, esophageal squamous cell carcinoma; HGIN, high‐grade intraepithelial neoplasia; HR, hazard ratio; LPM, lamina propria; LVL, Lugol‐voiding lesion; MM, muscularis mucosa.

We also analyzed the risk factors for second primary LC, which were significantly associated with the outcomes (Table 4). Univariate analysis revealed that it was associated with metachronous ESCC (*p* = 0.014). As multiple LVLs tended to be associated with SPMs in this study, and smoking, age and sex have been reported as risk factors for LC^24^, ^25^ and are thought to be important factors, multivariate analysis was performed including these factors. As a result, metachronous ESCC was a significant risk factor of second primary LC (*p* = 0.037, HR 3.51, 95% CI: 1.08–11.41), and age > 69 years old tended to relate be associated with it (*p* = 0.055, HR 2.90, 95% CI: 0.98–8.60).

**TABLE 4:** Univariate and multivariate analyses for second primary LC after ESCC resection.

	Second primary LC	Univariate analysis HR [95% CI]	*p* Value	Multivariate analysis HR [95% CI]	*p* Value
Age					
<70‐years‐old	7.1% (6/98)	1	0.330	1	0.055
70‐years‐old	10.1% (10/89)	1.36 [1.02–1.82]		2.90 [0.98–8.60]	
Sex					
Female	8.1% (3/37)	1	0.249	1	0.598
Male	8.7% (13/150)	0.80 [0.56–1.16]		0.70 [0.18–2.68]	
Smoking					
No	6.1% (2/33)	1	0.847	1	0.427
Yes	9.4% (13/138)	1.73 [0.39–7.67]		1.89 [0.39–9.10]	
Unknown	6.3% (1/16)				
Alcohol consumption					
No	11.1% (3/27)	1	0.800		
Yes	8.4% (12/143)	1.05 [0.70–1.60]			
Unknown	5.9% (1/17)				
Multiple LVLs					
No	2.8% (1/36)	1	0.103	1	0.323
Yes	10.0% (15/150)	3.95 [0.52–29.90]		2.90 [0.35–23.86]	
Unknown	0% (0/1)				
Location					
Upper	10.3% (3/29)	1	0.905		
Middle	8.0% (10/125)	0.74 [0.20–2.69]			
Lower	9.1% (3/33)	0.77 [0.16–3.82]			
Size					
<15 mm	7.2% (6/83)	1	0.510		
≥15 mm	9.6% (10/104)	1.41 [0.51–3.87]			
Macroscopic type					
Flat or depressed type	9.1% (16/175)	1	0.120		
Elevated or mixed type	0% (0/12)	–			
Pathological Invasion depth					
HGIN	11.0% (8/73)	1	0.287		
EP/LPM	8.0% (7/88)	0.95 [0.69–1.30]			
MM	3.9% (1/26)	0.82 [0.52–1.29]			
Simultaneous or history of cancer in other organs					
No	7.6% (10/129)	1	0.208		
Yes	10.4% (6/58)	1.23 [0.90–1.68]			
Metachronous ESCC					
No	5.8% (8/139)	1	0.014	1	0.037
Yes	16.7% (8/48)	3.80 [1.31–10.99]		3.51 [1.08–11.41]	

Abbreviations: CI, confidence interval; EP, epithelium; ESCC, esophageal squamous cell carcinoma; HGIN, high‐grade intraepithelial neoplasia; HR, hazard ratio; LC, lung cancer; LPM, lamina propria; LVL, Lugol‐voiding lesion; MM, muscularis mucosa.

### 3.5 Details of the second primary LC cases

Among the 16 patients with second primary LC, 81.3% (13/16) were smokers, and all nine patients who died due to LC were smokers (Table 5). Compared with the seven living patients with LC, nine dead patients were diagnosed after they had progressed to stages II–IV. The two patients who were initially diagnosed with stage I LC but died due to LC had developed metastatic recurrence after receiving LC treatment, then died because of it. Additionally, the interval between the chest CT scan when LC was initially detected and the prior CT scan during which LC failed to be detected was calculated as >1 year in 66.7% (6/9) of dead patients with LC and 16.7% (1/6) of live patients.

**TABLE 5:** Details and their outcomes of second primary LC cases diagnosed after ESCC resection.

	Age	Sex	LC *s*tage	LC histology	Smoking	CT before ESD	From the prior CT to LC (months)	From ESD to LC (months)	Survival or observation periods after LC (months)
LC death patients	77	M	I	Adenocarcinoma	Yes	Yes	13.2	15.3	34.6
	71	M	I	Adenocarcinoma	Yes	Yes	11.5	56.0	35.7
	75	M	II	SCC	Yes	Yes	12.1	48.0	34.5
	63	M	II	SCC	Yes	Yes	6.5	15.7	7.2
	66	M	II	SCC	Yes	Yes	18.2	29.3	0.4
	69	M	III	Unknown	Yes	Yes	70.5	107.6	1.8
	56	F	IV	Small cell carcinoma	Yes	Yes	14.1	13.1	13.5
	70	F	IV	Small cell carcinoma	Yes	Yes	3.7	42.5	32.9
	70	M	IV	Unknown	Yes	Yes	27.5	94.4	5.4
							Median 13.2 Range 3.7–70.5	Median 42.5 Range 13.1–107.6	Median 13.2 Range 3.7–70.5
LC alive patients	73	M	I	SCC	Yes	Yes	4.3	2.0	123.5
	66	M	I	SCC	Unknown	Yes	10.9	25.3	9.3
	73	F	I	Adenocarcinoma	Yes	No	10.5	70.2	45.4
	63	M	I	Adenocarcinoma	Yes	No	6.5	37.3	74.4
	74	M	I	Unknown	No	Yes	12.8	106.7	9.0
	57	M	III	Adenosquamous carcinoma	Yes	Yes	10.7	43.7	114.9
	72	M	IV	Adenocarcinoma	No	Yes	5.7	48.4	22.9
							Median 10.5 Range 4.3–12.8	Median 43.7 Range 2.0–106.7	Median 45.4 Range 9.0–123.5

Abbreviations: CT, computed tomography scan; ESCC, esophageal squamous cell carcinoma; ESD, endoscopic submucosal dissection; F, female; LC, lung cancer; M, male; SCC, squamous cell carcinoma.

## 4 DISCUSSION

In this study, we investigated the incidence of SPMs after curative treatment of superficial ESCC and the significantly poor prognosis of patients with SPMs. In addition, more than half of all deaths were due to malignancies, indicating the need for surveillance. As previously reported, the most commonly observed SPMs in patients with ESCC were HNSCC, LC, and gastric cancer,^14^, ^15^ and multiple LVLs were the important risk factor.^16^ To the best of our knowledge, this is the first study to show that SPMs, especially LC, which cannot be detected using EGD, have a strong impact on prognosis. Moreover, we found that occurring metachronous ESCC is the risk factor for second primary LC.

It is not surprising that SPMs that could not be detected by EGD were associated with a higher mortality rate. This is because no surveillance methods had been established based on any modality other than EGD after ESD. On the other hand, the malignancies that were detected by EGD could generally be detected in the early stage if EGD was appropriately performed and could be treated by minimally invasive treatment. The present study shows the extent by which the mortality rate differs between malignancies that can or cannot be detected by EGD after ESCC treatment. Although many SPMs, such as HNSCC or gastric cancer, were detected using EGD in this cohort, EGD surveillance for metachronous ESCC performed every 6–12 months enabled the early detection and treatment of these patients and improved their prognosis (Figure 2). Therefore, endoscopic surveillance would be appropriate not only for detecting metachronous ESCC but also for these cancers. In contrast, more than one‐third of the malignancies that could not be detected using EGD were the cause of death, and more than half of the patients with LC died of LC, suggesting delayed detection. Early‐stage LC was more frequent in live patients than in dead patients. We should remember the risk factors for LC and actively screen the patients after ESCC treatment.

The present study showed that the development of metachronous ESCC was significantly associated with the incidence of second primary LC. Although smoking is a widely known risk factor for LC,^26^, ^27^, ^28^ this study found no significant association between smoking and LC occurrence. One reason for this is the increased number of smokers (73.8%) among the study participants. However, more than 80% of patients with LC were smokers, and only two were nonsmokers. Among smokers, a second primary LC was more common in patients with metachronous ESCC (*p* = 0.012, HR 4.53, 95% CI: 1.39–14.72) (Figure S2). Therefore, LC should be increasingly considered when metachronous ESCC occurs in smokers. In addition, in this study, drinkers were significantly more likely to have multiple LVLs (*p* < 0.001) and metachronous ESCC (*p* = 0.009) than nondrinkers, as previously reported.^29^, ^30^, ^31^ A positive association between alcohol consumption and the risk of LC in smokers has been reported.^32^ Therefore, alcohol consumption may affect the incidence of LC in smokers. Other possible risk factors include the amount of smoking and alcohol consumption or continuous smoking or drinking after ESCC treatment. However, approximately 30% of information on these factors was missing in this retrospective study (Table S2); therefore, we could not analyze them. Considering the present results, we believe that regular chest screening is advisable during follow‐up for patients with metachronous ESCC, recognizing that they are at a high risk of developing a second primary LC.

Low‐dose CT scans, which can reportedly reduce LC mortality,^33^ are suitable for LC screening. Regarding chest screening before ESD, 77.5% (145/187) of the patients underwent a chest CT scan before ESD (Table 1). In addition, all the patients who had not undergone a CT scan underwent chest radiography, except five patients. All LC death patients in this study had a chest CT scan prior to ESD, but the follow‐up CT scan interval for approximately half of the cases exceeded >1 year. In contrast, many of the live patients with LC underwent CT scans every 6–12 months. Thus, we believe that high‐risk patients, such as smokers or/and those with metachronous ESCC during the follow‐up period, should undergo CT scans at least once a year or stricter screening is warranted.

This study had several limitations due to its retrospective nature. First, there was selection bias. As this was a multicenter study, bias was reduced. Second, some patients had a short follow‐up duration. The median follow‐up time was 96.8 months, and 77% of all patients were followed up for longer than 5 years; therefore, the observation period was considered sufficient in many cases. Third, surveillance methods, such as modalities or intervals, were not standardized. Future studies are needed to determine appropriate surveillance methods and timeframes. Finally, there was a lack of data on smoking and drinking statuses. Further prospective studies with a larger number of cases, longer follow‐up periods, and fewer missing values are warranted.

In conclusion, after curative resection of superficial ESCC, it is important to be aware of the risk of SPMs, especially those that cannot be detected using EGD, such as LC. Metachronous ESCC was significantly related to second primary LC. We suggest strict screening not only via EGD but also via CT scan for patients with such risk factors to detect SPMs, including LC, in their early stages.

## AUTHOR CONTRIBUTIONS

**Ayaka Tajiri:** Conceptualization (equal); data curation (equal); formal analysis (lead); investigation (lead); methodology (lead); project administration (equal); resources (lead); writing – original draft (lead). **Yoshiki Tsujii:** Conceptualization (equal); data curation (equal); project administration (equal); supervision (lead); writing – review and editing (lead). **Tsutomu Nishida:** Data curation (equal); writing – review and editing (supporting). **Takuya Inoue:** Data curation (equal); writing – review and editing (supporting). **Akira Maekawa:** Data curation (equal); writing – review and editing (supporting). **Shinji Kitamura:** Data curation (equal); writing – review and editing (supporting). **Shinjiro Yamaguchi:** Data curation (equal); writing – review and editing (supporting). **Akihiro Nishihara:** Data curation (equal); writing – review and editing (supporting). **Takuya Yamada:** Data curation (equal); writing – review and editing (supporting). **Hideharu Ogiyama:** Data curation (equal); writing – review and editing (supporting). **Yoko Murayama:** Data curation (equal); writing – review and editing (supporting). **Shunsuke Yamamoto:** Data curation (supporting); writing – review and editing (supporting). **Satoshi Egawa:** Data curation (supporting); writing – review and editing (supporting). **Ryotaro Uema:** Investigation (supporting); project administration (supporting); writing – review and editing (supporting). **Takeo Yoshihara:** Investigation (supporting); project administration (supporting); writing – review and editing (supporting). **Yoshito Hayashi:** Conceptualization (supporting); investigation (supporting); project administration (supporting); supervision (lead); writing – review and editing (lead). **Tetsuo Takehara:** Project administration (supporting); supervision (lead); writing – review and editing (lead).

## CONFLICT OF INTEREST STATEMENT

There is no conflict of interest to declare.

## ETHICS STATEMENT

The study protocol was approved by the Institutional Review Boards of Osaka University (no. 16352) and other participating institutions. The study was conducted in accordance with the principles of the Declaration of Helsinki.

## Supporting information


Figure S1.

Figure S2.

Data S1.

## Data Availability

The datasets generated and analyzed during the current study are available from the corresponding author on reasonable request.
