# Supplementary material for: High incidence of lung cancer death after curative endoscopic submucosal dissection for superficial esophageal squamous cell carcinoma
Source: Cancer Med. 2024 May 11;13(9):e7242. doi: 10.1002/cam4.7242 (PMC11087847; doi:10.1002/cam4.7242)
Supplement: Supplementary file 1 — Figure S1. [file CAM4-13-e7242-s001.pdf]

(A) (B)

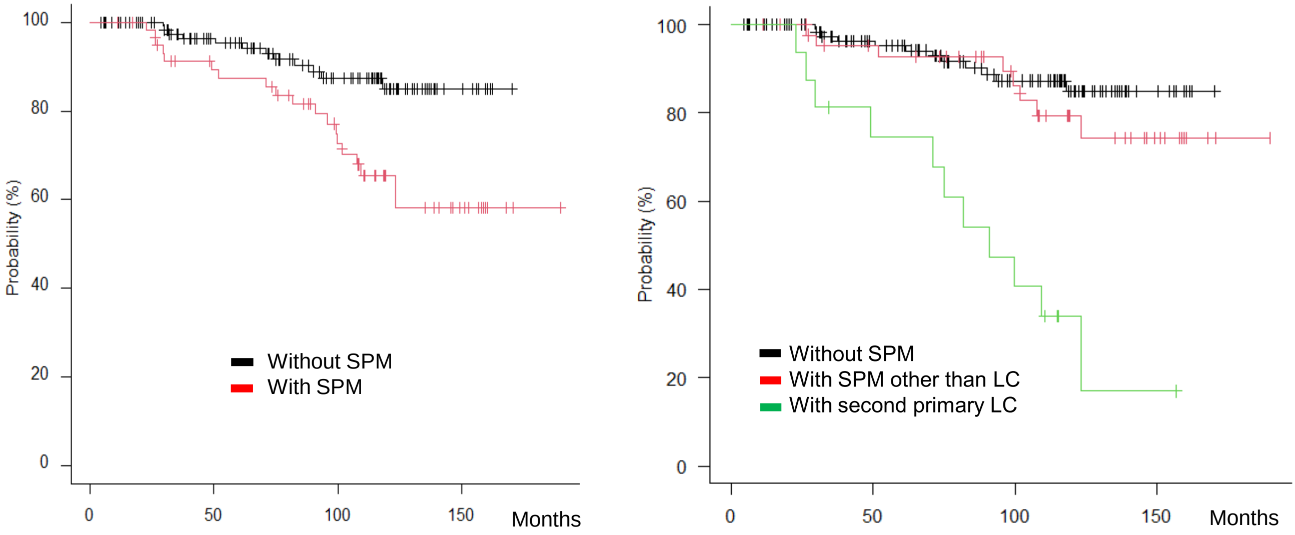

(A) Number at risk

Without SPM	127	91	55	9
With SPM	60	47	32	10

(B) Number at risk

Without SPM	126	90	55	9
With SPM other than LC	45	37	26	9
With second primary LC	16	11	6	1
