# Supplementary figures and images for: High incidence of lung cancer death after curative endoscopic submucosal dissection for superficial esophageal squamous cell carcinoma
Source: Cancer Med. 2024 May 11;13(9):e7242. doi: 10.1002/cam4.7242 (PMC11087847; doi:10.1002/cam4.7242)

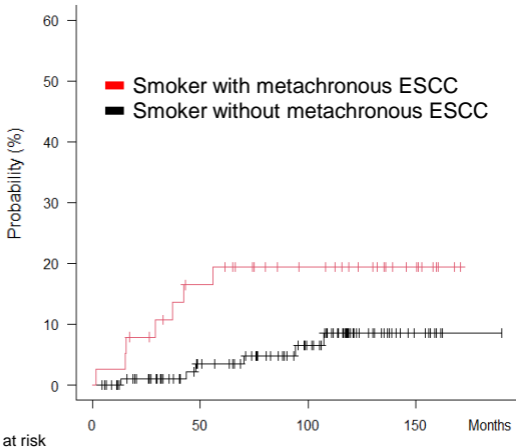

With metachronous  
ESCC

38

28

19

8

Without metachronous  
ESCC

100

69

45

6

Supplement: Supplementary file 2 — Figure S2. [file CAM4-13-e7242-s002.pdf]
