# Supplementary material for: High incidence of lung cancer death after curative endoscopic submucosal dissection for superficial esophageal squamous cell carcinoma
Source: Cancer Med. 2024 May 11;13(9):e7242. doi: 10.1002/cam4.7242 (PMC11087847; doi:10.1002/cam4.7242)
Supplement: Supplementary file 3 — Data S1. [file CAM4-13-e7242-s003.docx]

Table 1. Patient and lesion characteristics

| All patients | | | 187 |
| --- | --- | --- | --- |
| Age, median [range], years | | | 69 [49-92] |
| Sex | Male  Female | | 150 (80.2%)  37 (19.8%) |
| Alcohol consumption | Yes  No  Unknown | | 143 (76.5%)  27 (14.4%)  17 (9.1%) |
| Smoking | Yes  No  Unknown | | 138 (73.8%)  33 (17.6%)  16 (8.6%) |
| Simultaneous or history of cancer in other organs | | Yes  No | 58 (31.0%)  129 (69.0%) |
| Chest radiography before ESD | Yes  No  Unknown | | 158 (84.5%)  24 (12.8%)  5 (2.7%) |
| Chest CT scan before ESD | Yes  No  Unknown | | 145 (77.5%)  37 (19.8%)  5 (2.7%) |
| Lesions | | |  |
| Multiple LVLs | Yes  No  Unknown | | 150 (80.2%)  36 (19.3%)  1 (0.5%) |
| Tumor length, median [range], mm | | | 15 [2-85] |
| Location | Cervical-upper thoracic  Middle thoracic  Lower thoracic-abdominal | | 29 (15.5%)  125 (66.8%)  33 (17.6%) |
| Macroscopic type | Flat or depressed type (0-IIb,0-IIc)  Elevated (0-I, 0-IIa) or Mixed type | | 175 (93.6%)  12 (6.4%) |
| Pathological depth of invasion | HGIN  EP/LPM  MM | | 58 (31.0%)  103 (55.1%)  26 (13.9%) |
| Multiple ESCC | Yes  No | | 24 (12.8%)  163 (87.2%) |
| Metachronous malignancies | | |  |
| Observation time, median [range], months | | | 96.8 [4.6-192.7] |
| Metachronous ESCC | Yes  No | | 48 (25.7%)  139 (74.3%) |
| Second primary malignancy | Yes  No | | 60 (32.1%)  127 (67.9%) |

ESD, endoscopic submucosal dissection; CT, computed tomography; LVL, Lugol-voiding lesion; HGIN, high-grade intraepithelial neoplasia; EP, epithelium; LPM, lamina propria; MM, muscularis mucosa; ESCC esophageal squamous cell carcinoma.

Table 2. Association between SPM or second primary LC and their prognoses

|  |  | HR for mortality [95% CI] | *P* value |
| --- | --- | --- | --- |
| With SPM | No  Yes | 1  6.57 [3.10–13.93] | <0.001 |
| With second primary LC | No  Yes | 1  13.03 [6.13–27.67] | <0.001 |

SPM, second primary malignancy; LC, lung cancer; HR, hazard ratio; CI, confidence interval.

Table 3. Univariate and multivariate analyses for second primary malignancies after ESCC resection

|  |  | | Second primary malignancies | Univariate analysis  HR [95% CI] | *P* value | Multivariate analysis  HR [95% CI] | *P* value |
| --- | --- | --- | --- | --- | --- | --- | --- |
| Age | -69 years old  70- years old | | 32.7% (32/98)  31.5% (28/89) | 1  1.01 [0.61–1.70] | 0.955 | 1  1.31 [0.75–2.26] | 0.340 |
| Sex | Female  Male | | 27.0% (10/37)  33.3% (50/150) | 1  1.16 [0.69–2.29] | 0.666 | 1  0.85 [0.38–1.93] | 0.702 |
| Smoking | No  Yes  Unknown | | 27.3% (9/33)  36.2% (50/138)  6.3% (1/16) | 1  1.45 [0.71–2.94] | 0.360 | 1  1.27 [0.57–2.82] | 0.557 |
| Alcohol consumption | No  Yes  Unknown | | 33.3% (9/27)  35.0% (50/143)  5.9% (1/17) | 1  1.34 [0.66–2.74] | 0.416 | 1  1.22 [0.51–2.93] | 0.650 |
| Multiple LVLs | No  Yes  Unknown | | 13.9% (5/36)  36.0% (54/150)  100% (1/1) | 1  3.33 [1.33–8.36] | 0.010 | 1  2.43 [0.91–6.50] | 0.077 |
| Location | Upper  Middle  Lower | | 37.9% (11/29)  28.0% (35/125)  42.4% (14/33) | 1  0.61 [0.31–1.20]  0.96 [0.44–2.13] | 0.199 |  |  |
| Size | >15 mm  15 mm≦ | | 34.9% (29/83)  29.8% (31/104) | 1  0.90 [0.54–1.49] | 0.681 |  |  |
| Macroscopic type | Flat or depressed type  Elevated or mixed type | | 30.9% (54/175)  50.0% (6/12) | 1  1.68 [0.72–3.91] | 0.228 |  |  |
| Pathological Invasion depth | HGIN  EP/LPM  MM | | 35.6% (26/73)  29.6% (26/88)  39.8% (8/26) | 1  0.75 [0.44–1.30]  0.81 [0.37–1.79] | 0.591 |  |  |
| Simultaneous or history of cancer in other organs | | No  Yes | 29.5% (38/129)  37.9% (22/58) | 1  1.41 [0.83–2.42] | 0.216 | 1  1.20 [0.69–2.07] | 0.514 |
| Metachronous ESCC | No  Yes | | 24.5% (34/139)  52.1% (25/48) | 1  1.92 [1.05–3.53] | 0.035 | 1  1.60 [0.83–3.07] | 0.157 |

ESCC, esophageal squamous cell carcinoma; HR, hazard ratio; CI, confidence interval; HGIN, high-grade intraepithelial neoplasia; EP, epithelium; LPM, lamina propria; MM, muscularis mucosa; LVL, Lugol-voiding lesion.

Table 4. Univariate and multivariate analyses for second primary LC after ESCC resection

|  |  | | Second primary LC | Univariate analysis  HR [95% CI] | *P* value | Multivariate analysis  HR [95% CI] | *P* value |
| --- | --- | --- | --- | --- | --- | --- | --- |
| Age | -69 years old  70- years old | | 7.1% (6/98)  10.1% (10/89) | 1  1.36 [1.02–1.82] | 0.330 | 1  2.90 [0.98–8.60] | 0.055 |
| Sex | Female  Male | | 8.1% (3/37)  8.7% (13/150) | 1  0.80 [0.56–1.16] | 0.249 | 1  0.70 [0.18–2.68] | 0.598 |
| Smoking | No  Yes  Unknown | | 6.1% (2/33)  9.4% (13/138)  6.3% (1/16) | 1  1.73 [0.39–7.67] | 0.847 | 1  1.89 [0.39–9.10] | 0.427 |
| Alcohol consumption | No  Yes  Unknown | | 11.1% (3/27)  8.4% (12/143)  5.9% (1/17) | 1  1.05 [0.70–1.60] | 0.800 |  |  |
| Multiple LVLs | No  Yes  Unknown | | 2.8% (1/36)  10.0% (15/150)  0% (0/1) | 1  3.95 [0.52–29.90] | 0.103 | 1  2.90 [0.35–23.86] | 0.323 |
| Location | Upper  Middle  Lower | | 10.3% (3/29)  8.0% (10/125)  9.1% (3/33) | 1  0.74 [0.20–2.69]  0.77 [0.16–3.82] | 0.905 |  |  |
| Size | >15 mm  15 mm≦ | | 7.2% (6/83)  9.6% (10/104) | 1  1.41 [0.51–3.87] | 0.510 |  |  |
| Macroscopic type | Flat or depressed type  Elevated or mixed type | | 9.1% (16/175)  0% (0/12) | 1  - | 0.120 |  |  |
| Pathological Invasion depth | HGIN  EP/LPM  MM | | 11.0% (8/73)  8.0% (7/88)  3.9% (1/26) | 1  0.95 [0.69–1.30]  0.82 [0.52–1.29] | 0.287 |  |  |
| Simultaneous or history of cancer in other organs | | No  Yes | 7.6% (10/129)  10.4% (6/58) | 1  1.23 [0.90–1.68] | 0.208 |  |  |
| Metachronous ESCC | No  Yes | | 5.8% (8/139)  16.7% (8/48) | 1  3.80 [1.31–10.99] | 0.014 | 1  3.51 [1.08–11.41] | 0.037 |

LC, lung cancer; ESCC, esophageal squamous cell carcinoma; HR, hazard ratio; CI, confidence interval; LVL, Lugol-voiding lesion; HGIN, high-grade intraepithelial neoplasia; EP, epithelium; LPM, lamina propria; MM, muscularis mucosa.

Table 5. Details and their outcomes of second primary LC cases diagnosed after ESCC resection

|  | Age | Sex | LC *s*tage | LC histology | Smoking | CT before ESD | From the prior CT to LC (months) | From ESD to LC (months) | Survival or observation periods after LC (months) |
| --- | --- | --- | --- | --- | --- | --- | --- | --- | --- |
| **LC death patients** | 77 | M | I | Adenocarcinoma | Yes | Yes | 13.2 | 15.3 | 34.6 |
|  | 71 | M | I | Adenocarcinoma | Yes | Yes | 11.5 | 56.0 | 35.7 |
|  | 75 | M | II | SCC | Yes | Yes | 12.1 | 48.0 | 34.5 |
|  | 63 | M | II | SCC | Yes | Yes | 6.5 | 15.7 | 7.2 |
|  | 66 | M | II | SCC | Yes | Yes | 18.2 | 29.3 | 0.4 |
|  | 69 | M | III | Unknown | Yes | Yes | 70.5 | 107.6 | 1.8 |
|  | 56 | F | IV | Small cell carcinoma | Yes | Yes | 14.1 | 13.1 | 13.5 |
|  | 70 | F | IV | Small cell carcinoma | Yes | Yes | 3.7 | 42.5 | 32.9 |
|  | 70 | M | IV | Unknown | Yes | Yes | 27.5 | 94.4 | 5.4 |
|  |  |  |  |  |  |  | Median 13.2  Range 3.7–70.5 | Median 42.5  Range 13.1–107.6 | Median 13.2  Range 3.7–70.5 |
| **LC alive patients** | 73 | M | I | SCC | Yes | Yes | 4.3 | 2.0 | 123.5 |
|  | 66 | M | I | SCC | Unknown | Yes | 10.9 | 25.3 | 9.3 |
|  | 73 | F | I | Adenocarcinoma | Yes | No | 10.5 | 70.2 | 45.4 |
|  | 63 | M | I | Adenocarcinoma | Yes | No | 6.5 | 37.3 | 74.4 |
|  | 74 | M | I | Unknown | No | Yes | 12.8 | 106.7 | 9.0 |
|  | 57 | M | III | Adenosquamous carcinoma | Yes | Yes | 10.7 | 43.7 | 114.9 |
|  | 72 | M | IV | Adenocarcinoma | No | Yes | 5.7 | 48.4 | 22.9 |
|  |  |  |  |  |  |  | Median 10.5  Range 4.3–12.8 | Median 43.7  Range 2.0–106.7 | Median 45.4  Range 9.0–123.5 |

LC, lung cancer; ESCC, esophageal squamous cell carcinoma; CT, computed tomography scan; ESD, endoscopic submucosal dissection; M, male; F, female; SCC, squamous cell carcinoma.

Supplemental Table 1. The participating institutions.

| **The participating institutions** |
| --- |
| Osaka University Hospital |
| Toyonaka Municipal Hospital |
| Osaka General Medical Center |
| Osaka Police Hospital |
| Sakai City Medical Center |
| Kansai Rosai Hospital |
| Minoh City Hospital |
| Osaka Rosai Hospital |
| Ikeda City Hospital |
| Itami City Hospital |
| National Hospital Organization Osaka National Hospital |

Supplemental Table 2. Supporting data about patient characteristics

| All patients | | 187 |
| --- | --- | --- |
| Amount of alcohol consumption before ESD | Never/ rare  Light  Moderate  Heavy  Unknown | 26 (13.9%)  39 (20.9%)  26 (20.9%)  44 (23.5%)  52 (27.8%) |
| Amount of smoking before ESD (Brinkman index) | Median [range] | 900 [10-5760] |
| Alcohol consumption after ESD | Yes  No  Unknown | 61 (32.6%)  51 (27.3%)  75 (40.1%) |
| Smoking after ESD | Yes  No  Unknown | 25 (13.4%)  108 (57.8%)  54 (28.9%) |

ESD, endoscopic submucosal dissection; Alcohol consumption never/rare, consumed alcohol <1 U/week; light, 1–8.9 U/week; moderate, 9–17.9 U/week; heavy, ≥18 U/week; Brinkman index = (number of cigarettes smoked per day) × (number of years smoked).
